# Low mechano-afferent fibers reduce thermal pain but not pain intensity in CRPS

**DOI:** 10.1186/s12883-021-02304-7

**Published:** 2021-07-09

**Authors:** Kathrin Habig, Gothje Lautenschläger, Hagen Maxeiner, Frank Birklein, Heidrun H. Krämer, Susann Seddigh

**Affiliations:** 1 Department of Neurology, Justus Liebig University, 35392 Giessen, Germany; 2 Department of Anaesthesiology, Justus Liebig University, 35392 Giessen, Germany; 3 Department of Neurology, University Medical Center, Johannes Gutenberg-University, Langenbeckstr. 1, 56101 Mainz, Germany; 4 Department of Neurology, BG Klinikum Duisburg, 47249 Duisburg, Germany

**Keywords:** CT afferents, CRPS, Pleasantness of touch, Pain modulation, Allodynia, QST

## Abstract

**Background:**

Human hairy (not glabrous skin) is equipped with a subgroup of C-fibers, the C-tactile (CT) fibers. Those do not mediate pain but affective aspects of touch. CT-fiber-activation reduces experimental pain if they are intact. In this pilot study we investigated pain modulating capacities of CT-afferents in CRPS.

**Methods:**

10 CRPS-patients (mean age 33 years, SEM 3.3) and 11 healthy controls (mean age 43.2 years, SEM 3.9) participated.

CT-targeted-touch (brush stroking, velocity: 3 cm/s) was applied on hairy and glabrous skin on the affected and contralateral limb. Patients rated pleasantness of CT-targeted-touch (anchors: 1 “not pleasant”—4 “very pleasant”) twice daily on 10 days. Pain intensity (NRS: 0 “no pain” – 10 “worst pain imaginable”) was assessed before, 0, 30, 60 and 120 min after each CT-stimulation. To assess sensory changes, quantitative-sensory-testing was performed at the beginning and the end of the trial period.

**Results:**

CT-targeted-touch was felt more pleasant on the healthy compared to the affected limb on hairy (p < 0.001) and glabrous skin (p 0.002), independent of allodynia. In contrast to healthy controls patients felt no difference between stimulating glabrous and hairy skin on the affected limb. Thermal pain thresholds increased after CT-stimulation on the affected limb (cold-pain-threshold: p 0.016; heat-pain-threshold: p 0.033).

**Conclusions:**

CT-stimulation normalizes thermal pain thresholds but has no effect on the overall pain in CRPS. Therefore, pain modulating properties of CT-fibers might be too weak to alter chronic pain in CRPS. Moreover, CT-fibers appear to lose their ability to mediate pleasant aspects of touch in CRPS.

## Background

Complex regional pain syndrome (CRPS) occurs in 2–5% of patients [1] suffering from relevant trauma, e.g. fractures, but can also develop after minor injuries. CRPS can be divided regarding the presence of a nerve lesion (CRPS I without nerve lesion; CRPS II with proof of a nerve lesion). The diagnostic revised Budapest criteria [2] include reported symptoms and clinical signs at the time of the investigation in the affected region. In particular, pain and hyperalgesia, sensory, vasomotor, sudomotor/edema and motor/trophic symptoms are assessed. Clinically, there is no difference between CRPS I and II and electrophysiological tests cannot always detect nerve lesions of small nerve branches.

Quantitative sensory testing in patients with CRPS expounded that sensory abnormalities are individually very different ranging from loss to gain of function for the same modality tested [3]. Ott and coworkers found a decreased sensitivity for touch and an increased sensitivity to pinprick stimuli for nearly half of the investigated CRPS patients (CRPS I and II) and defined spontaneous pain sensations and pain augmentation of any cause as positive predictors for CRPS [4]. A higher sensitivity to pin prick pain and blunt pressure in CRPS patients compared to patients with “normal” fractures of the upper limb has been observed [5]. J. Gierthmühlen and associates presented that patients with CRPS showed heat (36–44%) or pressure hyperalgesia (67–73%) as well as thermal hypoesthesia (30–44%). Especially for CRPS I patients, hyperalgesia and allodynia without loss of small fiber function were more frequent than in CRPS II or patients with peripheral nerve injury [6].

These findings point to a disinhibition of nociception in CRPS patients, whereas the large sensory fibers are intact (normal detection thresholds).

CT afferents are a subgroup of C fibers, the C-low threshold mechanoreceptive (C-LTMR; CT = C touch or C tactile) afferents. Together with Aβ afferents, CT fibers mediate pleasant aspects of touch in human hairy skin. CT fibers were first detected in humans in the infraorbital nerve by microneurography [7] and have later been detected in hairy skin of arms and legs [8]. They could not be found in glabrous skin [8, 9]. The C-LTMRs follow the lamina I/II spinothalamic pathway to the ventromedial posterior thalamic nucleus [10] and from there to the contralateral posterior insular cortex [11] and the medial prefrontal cortex [12]. Microneurographic studies of C-LTMRs characterized these afferents as reacting to a low mechanical indentation force (< 5 mN) [13] and to slowly moving stimuli (intermediate velocities of 1-10 cm/s) [14, 15], which resamples gentle stroking. They seem to be a main signaling path for pleasantness of touch, since pleasantness of touch is correlated with the firing rate of CT fibers [15]. Seal and Nagi proposed, that low threshold mechanosensitive afferents contribute to experimentally induced allodynia [16, 17]. Another study demonstrated that perception of allodynia is associated with impaired processing of affective touch by investigating patients with a rare hedonic loss of Aβ afferents, pointing to a linkage of CT afferents and allodynia [12].

In addition, recent studies reported that CT stimulation can reduce experimental pain in healthy individuals [18]. In pathophysiological conditions (e.g. small fiber neuropathy (SFN) patients with a reduced intraepidermal nerve fiber density (IENFD) implying a corresponding reduction of CT afferents) gentle stroking loses its pain modulating properties [19]. Prior publications have examined IENFD in CRPS patients and reported contradicting results [20, 21]. Recent investigations of our group did not detect reduced IENFD in CRPS. And also QST profiles detected small fiber dysfunction only in 39% of CRPS I patients versus 48% with large fiber dysfunction [6]. On the other hand Caty and coworkers detected a dysfunction of C and A-Delta fibers (small diameter fibers) in CRPS I patients performing laser evoked potentials [22].

These observations gave rise to the question whether CT afferents have an impact on pain intensity as well as perception of allodynia or hyperalgesia in CRPS patients.

## Methods

### Participants

10 patients with CRPS (6 women, 4 men; mean age: 33 years SEM 3.3; age range: 16– 54 years) as well as 11 healthy controls (8 women, 3 men; mean age 43.2 years SEM 3.8; age range: 16– 63 years) were included in this pilot study.

All participants gave their informed written consent according to the latest revision of the Declaration of Helsinki. The study was approved by the Rhineland–Palatinate Medical Association (registration number 4208).

In all participants, a medical history was acquired, and a thorough neurological examination was undertaken. CRPS Type I and II were diagnosed according to the revised Budapest diagnostic criteria, scientific version [2]. In brief, all patients reported at least one symptom from 3 out of 4 categories: sensory, vasomotor, sudomotor / edema and motor / trophic. Additionally, at least two clinical signs needed to be present at the time of the investigation in two or more categories and there was no other diagnosis that better explained the signs and symptoms. CRPS patients with symptoms for 1 to 24 months were included, the mean duration of CRPS was 9.6 months (SEM 2.8). 5 patients reported allodynia. The presence of allodynia is important since tactile stimulation was performed on the affected limb. CRPS II has been confirmed in 3 patients (for further details see Table 1). An overview of the ongoing medication and multimodal pain therapy of each participant can be retraced in Table 2.

**Table 1:** Demographic data of all CRPS patients

**patient number**	**age [years]**	**sex [1 = female, 2 = male]**	**ADS**	**PDI**	**PESa**	**PESs**	**MPSS [1-4]**	**Von Korff [1-4]**	**NRS mean [0–10]**	**NRS min** **[0–10]**	**NRS max [0–10]**	**allodynia [1 = yes, 0 = no]**
1	54	2	12	41	20	14	1	3	5	4	8	0
2	34	2	20	34	38	37	2	4	6	3	8	0
3	29	1	26	43	28	27	3	4	6	4	8	1
4	21	1	32	39	29	32	2	3	7	4	9	0
5	31	1	33	53	46	35	3	4	7	6	10	1
6	36	1	47	60	56	18	3	4	8	7	10	0
7	38	1	18	47	47	40	3	4	8	4	10	1
8	38	2	46	66	53	33	3	4	7	7	10	1
9	16	1					2	4	8	7	9	1
10	33	2	11	40	17	28	3	4	5	1	8	0
**patient number**	**cause of CRPS**	**surgery [n]**	**duration [month]**		**Budapest criteria anamnesis [n/5]**		**Budapest criteria investigation [n/5]**		**CRPS type**	**affected limb [1 = arm, 2 = leg]**		**affected side [1 = right, 2 = left]**
1	fracture	1	3		3		3		1	2		1
2	contusion	1	10		4		5		2	1		2
3	reconstr. of ligaments	1	5		4		4		1	2		1
4	fracture	1	18		4		3		1	1		2
5	cyst resection	1	24		3		3		1	1		1
6	tendovaginitis sten	4	24		4		4		2	1		1
7	distorsion	4	1		4		3		2	2		2
8	fracture	4	2		2		5		1	1		2
9	contusion	0	3		5		4		1	1		1
10	contusion	3	4		5		5		1	1		2

*NRS* numeric rating scale, anchors 0 (no pain) to 10 (worst pain imaginable)

**Table 2:** Overview of medication prescribed during the investigation

patient number	COX inhibitors	opioids	Local/regional anaesthesiol	Tricykl AD	Atyp AD	Na Channel blockade	Ca Channel blockade	Cortisone	Alendronate	others
1			2 × IB	↑				C +	10 mg	
2	E↑		2 × PB	↑						Lidocain patch + , Capsaicin +
3	I↑									
4			4 × PB	Am + , ×						
5	Met →	MSI↓ ×	1 × Me, 1 × PA infiltration M. infraspinatus, M. teres minor, M. trapezius	D↓ ×	Du↓, M↓, V +	Ox +	Pre →			Clonidin → Fexofenadin → Zopiclon
6	Met →	MSI × Pa +	1 × PB Me		Du → , M →	Ox +	Pre →			
7	A +	MSI↓	1 × IB	Am →			Pre →			Lidocain patch + , Capsaicin + ×
8	Met↓, E +	Pal × Pa +	2x Infiltration M. infraspinatus 1 × PB, Me	Am↑	Du↑		Pre↑	C +	10 mg	
9	E → , D + , × , Met + , F + , ×		infiltration M. infraspinatus, SB, PB, Me KI	Am↑			Pre +			Lidocain patch +
10	I →		3 × SB	Am +				C +		

Overview of medication prescribed during the investigation. Arrows show increase↑, decrease ↓ or constant → medication doses. × medication stopped; + new medication. PB brachial plexus blockade, IB ischiadicus blockade, SB stellatum blockade, Me Mepivacain infusion, E Etoricoxib, I Ibuprofen, Met Metamizol, D Diclofenac, F Flupiritin, Am Amitriptylin, D Doxepin, M Mirtazapin, Du Duloxetin, V Venlafaxin, Ox Oxcarbazepin, Pre Pregabalin

For healthy volunteers (n = 11) the medical history was uneventful in all participants and the clinical neurological examination was normal.

### Quantitative sensory testing

We performed quantitative sensory testing on hairy skin of the affected and non-affected limb (dorsum of hand or foot) in every patient before and after performing CT stimulation, following the protocol of the German Research Network on Neuropathic Pain (DFNS) [23]. The detailed procedures are described elsewhere [23].

In brief, warm (WDT) and cold detection thresholds (CDT) were determined using a TSA 2001-II (MEDOC, Israel; baseline temperature of 32 °C; contact area of the thermode 9 cm^2^; ramp rate 1 °C/second). The threshold for cold and warm detection was calculated as mean of 4 consecutive measurements. Heat (HPT) and cold pain thresholds (CPT) were obtained by estimating the mean of 3 consecutive measurements. Thermal sensory limen (TSL) was assessed by alternating warm and cold stimuli (three times each), TSL was calculated as mean of the temperature differences. Participants were prompted to signal perceived temperature changes and were asked about paradoxical heat sensations (PHS). For mechanical pain thresholds (MPT) sets of calibrated pinpricks with a 0.25 mm flat-top cylindrical tip and a series of 7 forces (8–512 mN), geometrically spaced by a factor of 2 (The PinPrick, MRC systems, Heidelberg, Germany) were used. Mechanical pain sensitivity (MPS) and dynamic mechanical allodynia (DMA) was assessed by pain rating (range 0–100) of 50 randomized stimuli in blocks of 10 stimuli, each block consisting of 7 pinprick and 3 tactile moving stimuli. MPS was calculated as mean of all pain ratings assessed during pinprick stimuli. Dynamic mechanical allodynia (DMA) was calculated as geometric mean of all numerical pain ratings by tactile moving stimuli.

QST has been performed by a single trained investigator (SS) following the standardized verbal instructions and protocol provided by the German Research Network on Neuropathic Pain (DNFS) [23].

### Questionnaires

For the CRPS patients ADS (german general depression score “Allgemeine Depressionsskala”, based on the CES-D-Scale) [24], PESa and PESs (pain experience scale affective and sensory) [25], PDI (pain disability index) [26], MPSS (Mainz Pain staging system) [27] and von Korff Index [28] were assessed.

Subjects with ADS scores higher or equal the value 23 were categorized as “with depressive symptoms”. MPSS 1–3 assesses levels of chronic pain (1 = low pain chronification, 3 = high chronification). Von Korff Index distinguishes the degree of handicap (1 = low and 4 = high level of handicap). PDI is a questionnaire with 7 questions about impairment of daily life activities ranging from 0 (no impairment) to 10 (total impairment).

### Tactile stimulation

Repetitive stroking with a soft painter’s brush (2 cm wide, stimulation force 0.8 N; stimulation velocity 3 cm/sec) was applied in proximal to distal direction on the hairy part of the affected limb and the contralateral side (CT targeted touch) as well as on glabrous skin (e.g. sole of the foot or palm of the hand). In healthy individuals stroking has been performed on the foot (dorsum and sole of the foot). To avoid inter-investigator variability stroking was always performed by the same investigator (SS).

**Fig. 1:**
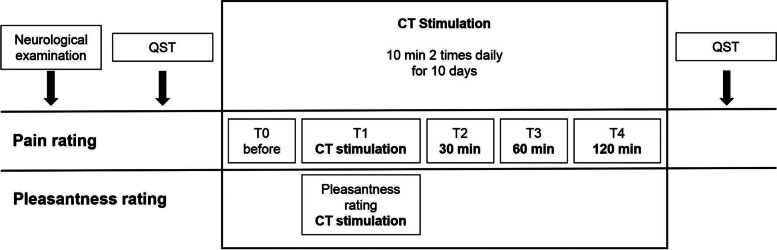
Illustration of the study protocol on a timescale. Shown are the onsets of pain rating (T0-T4) and pleasantness rating as well as the times of clinical and QST investigation

Tactile stimulation was performed over a period of 10 days, twice daily (each trial lasting 10 min) in each patient. For visualization of the study sequence see Fig. 1.

### Pain and pleasantness rating

The participants were asked to quantify their acute pain before (T0), during (T1), 30 min (T2), 60 min (T3) and 120 min (T4) after CT stimulation. To obtain comparable datasets pain intensity ratings at T0 (NRS(T0)) were set as 10, the proceeding ratings were estimated by multiplying with the quotient 10/NRS(T0). For better visualization of the results all parameters were divided by 10 afterwards.

During CT stimulation, the participants were asked to rate the pleasantness of CT targeted touch. Pleasantness was rated with 4 numbers: 4: very pleasant, 3: pleasant, 2: slightly pleasant, 1: not pleasant to achieve reliable and distinct answers. Rating of “1” included the sensation of unpleasantness. This has been explained to all participants beforehand in detail. In addition, patients were asked to evaluate their pain intensity on a numeric rating scale with the anchors 0: no pain and 10: worst pain imaginable (see Fig. 1 for the points of pain rating).

### Statistical analysis

Psychophysical data were analyzed using the SPSS Statistics (IBM, Version 23.0 for Windows) software package. Kolmogorov–Smirnov tests of normality were run for all data sets to evaluate normal distribution. If normal distribution was confirmed (pain rating) parametric statistics like two-sample or one sample t-tests were performed as indicated in the experiment-specific results. If normal distribution could not be assumed (pleasantness rating) non-parametric tests like Mann–Whitney-U test was performed. All values are given as mean ± standard error of mean (SEM) and standard deviation (SD) or as median and interquartile range (IQR). Values were considered significant if p $<$ 0.05.

Differences of pain ratings at all points of investigation (T0 to T4) were analysed using a one-way ANOVA.

QST parameters were log transformed (except PHS, CPT, HPT) and then transformed into z-scores (except PHS and DMA) using gender and age matched normative data published by [23] using the following expression: z-score = (X_single patient_—Mean_controls_)/SD_controls_ [29]. Z-scores higher than 2 indicate gain of function and lower than 2 loss of function sensory disturbances. PHS and DMA are given as absolute values (PHS 0–3, counting the number of paradoxical heat sensations during three trials; DMA NRS 0–100, according to the geometric mean of pain ratings of repetitive innocuous tactile stimuli).

For statistical comparison of group data we performed paired t-tests for each QST parameter (affected vs unaffected limb and 1^st^ vs 2^nd^ QST). Supplementary a method demonstrated by Magerl and coworkers for statistical comparison of QST group data was utilized. This method compares mean QST z-scores of a patient group with an ideal virtual sample with a mean z-score of “0” and a standard deviation of “1” utilizing a two sided independent t-test [30]. The test was performed using SISA online test for statistical analysis (www.quantitativeskills.com/sisa/statistics/t-test).

To demonstrate abnormal z-scores on the individual level, we assessed all absolute abnormal z-scores per QST parameter (results are given in percentage of the patient group) and abnormal side-to-side differences of each QST parameter (difference of more than 2 SDs retrieved from the reference data group in z-scores between affected and control limb) according to Gierthmühlen and coworkers [6].

## Results

### Questionnaires

ADS has been assessed in 9 patients (one patient is only 16 years of age, the scores are not validated for children). 5 patients rated a sum score ≥ 23 and were classified as “with depressive symptoms”.

All other scores were assessed to characterize the participants, these scores did not enter the statistics, for details see Table 1.

### Quantitative sensory testing

**Fig. 2:**
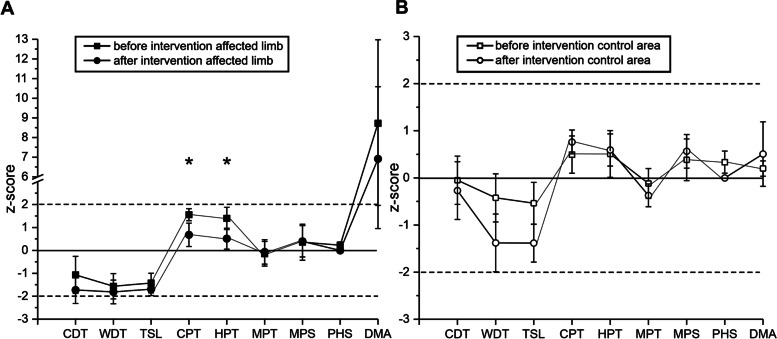
Quantitative sensory testing of CRPS patients is shown as z-Scores before (squares) and after the intervention (circles) at the affected limb (black symbols, **A**) and a control area (white symbols, **B**). 95% confidence interval resamples z-score ranging from -2 to 2. CDT: cold detection threshold, WDT: warm detection threshold, TSL: thermal sensory limen, CPT: cold pain threshold, HPT: heat pain threshold, MPS: mechanical pain sensitivity, MPT: mechanical pain threshold, PHS: paradoxical heat sensations, DMA: dynamic mechanical allodynia

The QST assessment on the affected limb revealed pathological z scores only for dynamic mechanical allodynia at both given points of testing (DMA affected limb: 1^st^ QST z-score = 9.56, SD 12.3; 2^nd^ QST z-score = 6.92, SD 10.998). QST on the control limb showed no pathological parameters regarding the z-scores at group level. For details see Fig. 2.

#### Comparison of each parameter on the affected and the unaffected limb (group level, z-scores)

At the time of the first QST assessment cold pain hyperalgesia was felt significantly more prominent on the affected limb (CPT affected limb: mean 1.62, SD 0.76, SEM 0.24; CPT control area: mean 0.6, SD 1.164, SEM 0.37; p 0.007, paired t-test). Hyperalgesia to heat pain showed the same trend (HPT affected limb: mean 1.64, SD 1.56, SEM 0.49; control area: mean 0.48, SD 1.41, SEM 0.45; p 0.081, paired t-test).

DMA on the affected limb was also significantly more pronounced than on the contralateral limb (affected limb: mean 9.56, SD 12.3, SEM 3.89; control limb: mean -0.04, SD 0.47, SEM 0.15; p 0.032, paired t-test). All other parameters showed no difference between the test (affected limb) and control area (unaffected limb).

The second QST revealed a significant difference comparing the investigation of the affected versus the unaffected limb regarding cold detection threshold (CDT affected limb: mean -1.71, SD 1.77, SEM 0.59; CDT control area: mean -0.26; SD 1.84; SEM 0.61; p 0.029, paired t-test). All other parameters displayed no difference between the test (affected limb) and control area (unaffected limb). Especially thermal pain thresholds and DMA aligned compared to the first QST.

#### Comparison of each parameter before (1^st^ QST) and after multimodal pain therapy and CT stimulation (2^nd^ QST) at group level, z-scores

Thermal pain thresholds (CPT and HPT) aligned to thresholds, provided by healthy individuals, at the affected limb at 2nd QST (1^st^ CPT: mean 1.56, SD 0.78, SEM 0.26; 2^nd^ CPT: mean 0.68, SD 1.53, SEM 0.51; p 0.016, paired t-test. 1^st^ HPT: mean 1.41, SD 1.47, SEM 0.49; 2^nd^ HPT: mean 0.52, SD 1.38, SEM 0.46; p 0.033, paired t-test). All QST parameter assessed on the control limb showed no significant difference over time. For Details see Fig. 2.

#### Group comparison of z-scores of each QST parameter from our patient group and an ideal virtual control group

In the first QST assessment of the affected limb, thermal detection and pain thresholds except cold detection threshold and DMA differed significantly from an ideal virtual group (WDT p 0.031; TSL p 0.015; CPT p 0.001; HPT p 0.016; DMA p 0.037; two-sided independent t-test). QST data of the control limb showed no significant difference compared to the ideal virtual sample. The second QST again showed significant abnormal thermal detection thresholds (CDT p 0.028; WDT p 0.018; TSL p 0.002), whereas thermal pain thresholds and perception of allodynia aligned to normal values. TSL of the control limb was also significantly different to the ideal sample (p 0.021).

#### Absolute abnormal z-scores and side-to-side difference (individual data)

**Fig. 3:**
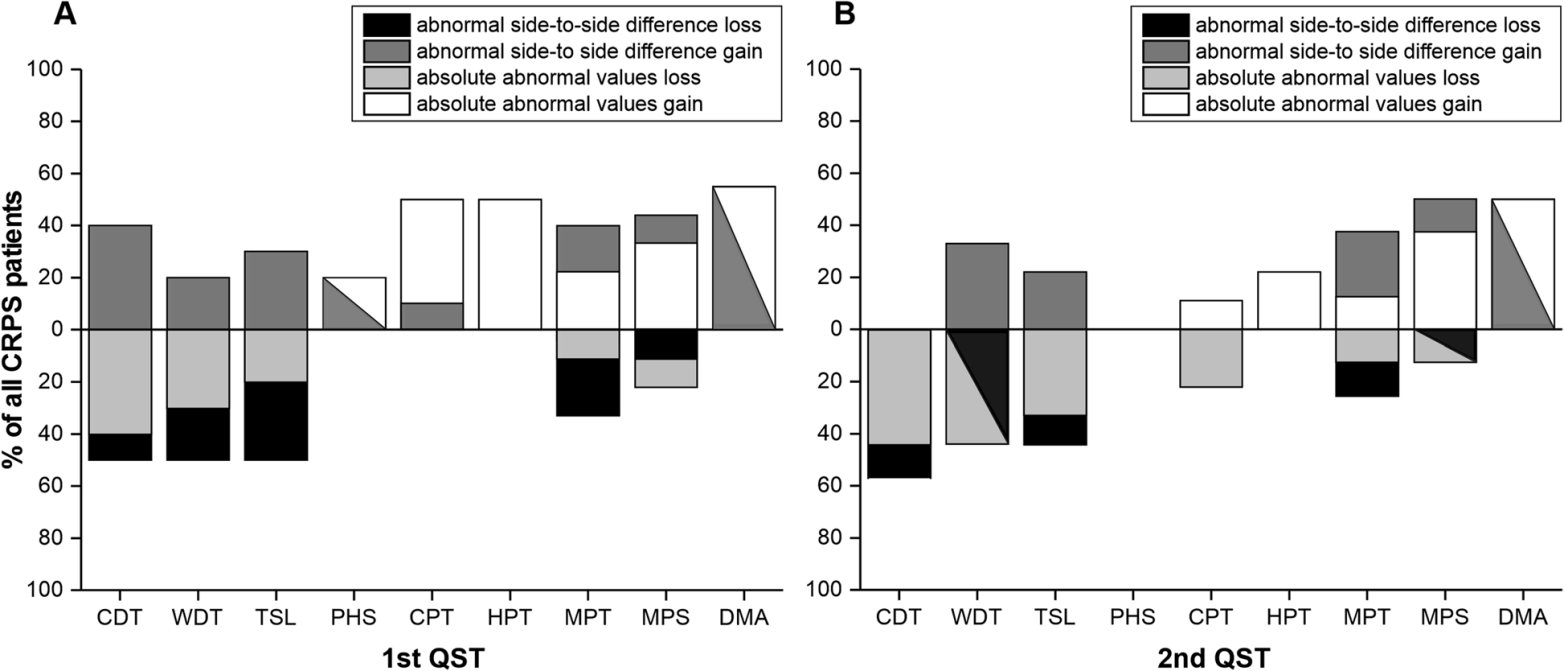
Loss and gain of function for absolute abnormal values and abnormal side-to-side differences of Quantitative sensory testing is shown for the first (**A**) and second (**B**) assessment on an individual level (graph shows percentage of abnormal values from all investigated patients)

For most QST data absolute abnormal z-scores revealed “plus signs” on the affected limb in our patient group (CDT, WDT, TSL, PHS, CPT and DMA; 1^st^ QST n = 10; 2^nd^ QST n = 9, except for MPS, MPT and DMA, here one participant refused the investigation at 1^st^ and 2^nd^ QST). Analysis of MPS and MPT showed positive and negative signs. Absolute abnormal negative z-scores (negative signs) could be retrieved from all individuals for assessment of heat pain threshold (HPT). See also Fig. 3.

Evaluation of abnormal side-to-side difference from the affected and the unaffected limb for each participant (“abnormal” defined as difference > 2 × SD for z-scores within normal range) loss and gain of function for CDT, WDT, TSL, MPS and MPT could be observed. Only gain of function could be observed for CPT, HPT, PHS and DMA. See also Fig. 3.

### Pleasantness ratings

Kolmogorov–Smirnov test was significant (p < 0.001), therefore non-parametric tests have been performed.

#### Healthy individuals

**Fig. 4:**
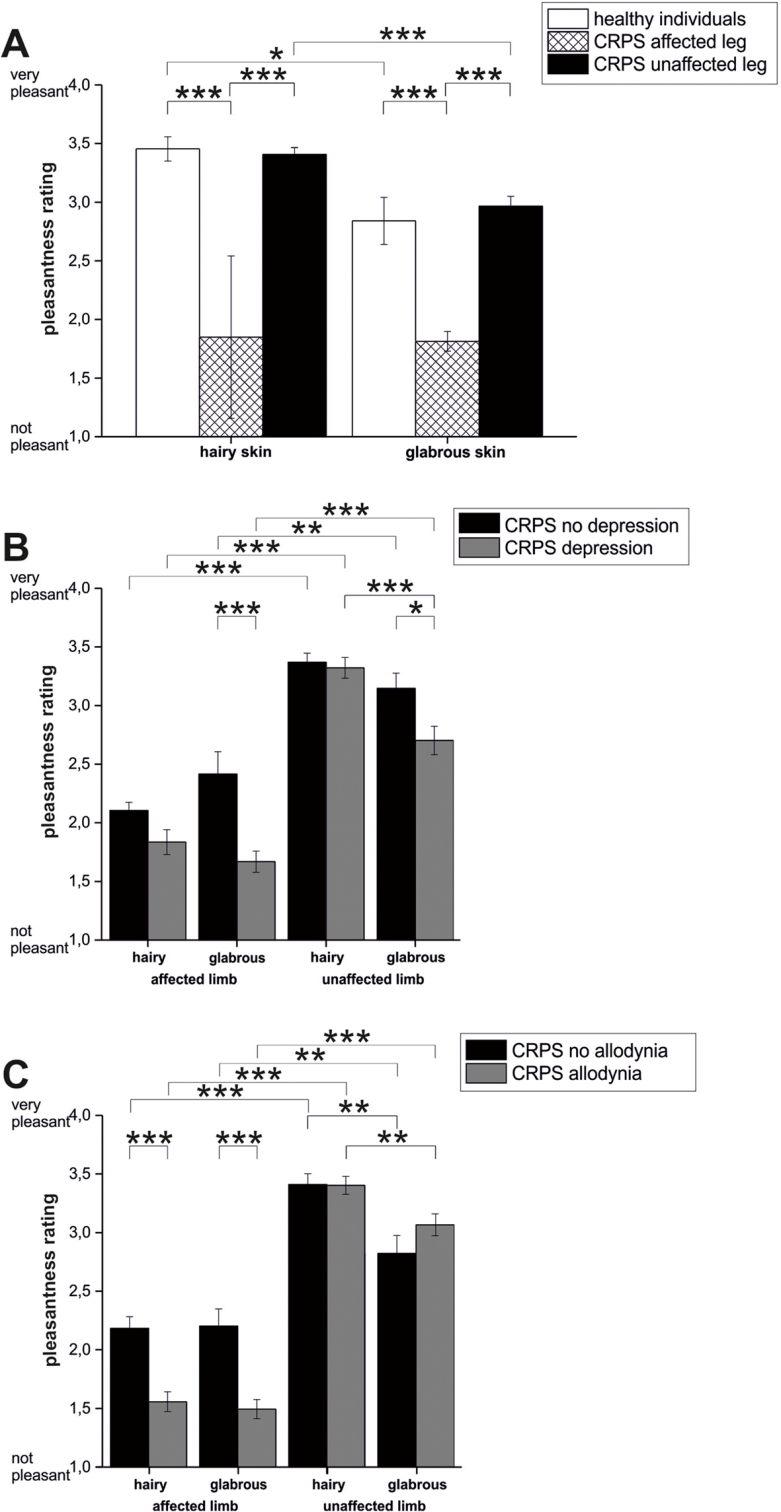
**A**: Pleasantness rating (anchors 1–4; 1 = not pleasant; 4 = very pleasant) for controls (healthy individuals: white) and CRPS patients (affected limb: black plaid on white; unaffected limb: black) by CT stimulation on hairy (left) and glabrous skin (right). **B**: Pleasantness rating for CRPS patients regarding depressive symptoms (no “depression”: black; “depression”: grey) for the affected limb (left) and unaffected limb (right). **C**: Pleasantness rating for CRPS patients regarding allodynia (no allodynia: black; allodynia grey) for the affected limb (left) and unaffected limb (right)

CT targeted touch was significantly more pleasant on hairy skin than on glabrous skin (hairy skin: median 3.25, IQR 1.0; glabrous skin: median 2.25, IQR 2.0; Mann–Whitney-U test, U = 151.0, p 0.024, n = 22). For details see Fig. 4A and B.

#### CRPS patients

Pleasantness of CT targeted touch was not significantly different between hairy (CT fibers present) or glabrous skin on the affected limb (hairy skin: median 2.0, IQR 1.5; glabrous skin: median 1.0, IQR 1.13; U = 9,713.5, p 0,74, n = 146). Stroking at the unaffected limb was significantly more pleasant on hairy skin (pleasantness rating median 4.0, IQR 1.0) than on glabrous skin (pleasantness rating median3.0, IQR 2.0; U = 5,276.5, p < 0.001, n = 144). CT targeted touch was significantly more pleasant on the contralateral limb than on the affected limb on hairy (U = 18,938,0, p < 0.001) and glabrous skin (U = 11,729.0, p < 0.001). See Fig. 4A.

#### Depression Scores

There were significant differences of pleasantness ratings comparing CRPS patients with and without depressive symptoms while CT stimulation on glabrous skin of both limbs (affected limb, with “depression”: median 1.0, IQR 1.0; without “depression” median 2.0, IQR 2.75; U = 1,092.0, p 0.001; contralateral limb, with “depression”: median 3.0, IQR 1.0; without “depression” median 3.0, IQR 1.5; U = 640.5, p 0.019, n = 85). For details see Fig. 4B.

#### Allodynia

CT targeted touch was less pleasant on the affected limb than on the contralateral limb regardless of stroking hairy or glabrous skin for patients with allodynia (hairy skin with allodynia, affected limb: median 1.0, IQR 1.0; unaffected limb median 4.0, IQR 1.0; glabrous skin with allodynia, affected limb: median 1.0, IQR 1.0; unaffected limb median 3.0, IQR 1.0; hairy skin U = 5,654.0, p < 0.001; glabrous skin U = 4,340.0, p < 0.001, n = 78). See Fig. 4C.

Patients suffering from allodynia rated CT targeted touch as less pleasant than patients without allodynia on the affected limb (hairy skin U = 1,557.5 p < 0.001; glabrous skin U = 1,612.5, p < 0.001) but not on the contralateral limb. In patients with allodynia stroking the unaffected limb on hairy skin was more pleasant then stroking glabrous skin (U = 1,758.0, p 0.006). For details see Fig. 4C.

CRPS patients without allodynia showed significant lower pleasantness ratings of gentle stroking on the affected limb (hairy skin: U = 1,557.5, p < 0.001; glabrous skin: U = 1,612.5, p < 0.001) but not on the contralateral limb. For details See Fig. 4C.

### Pain ratings

Kolmogorov–Smirnov test was not significant and parametric tests have been performed.

**Fig. 5:**
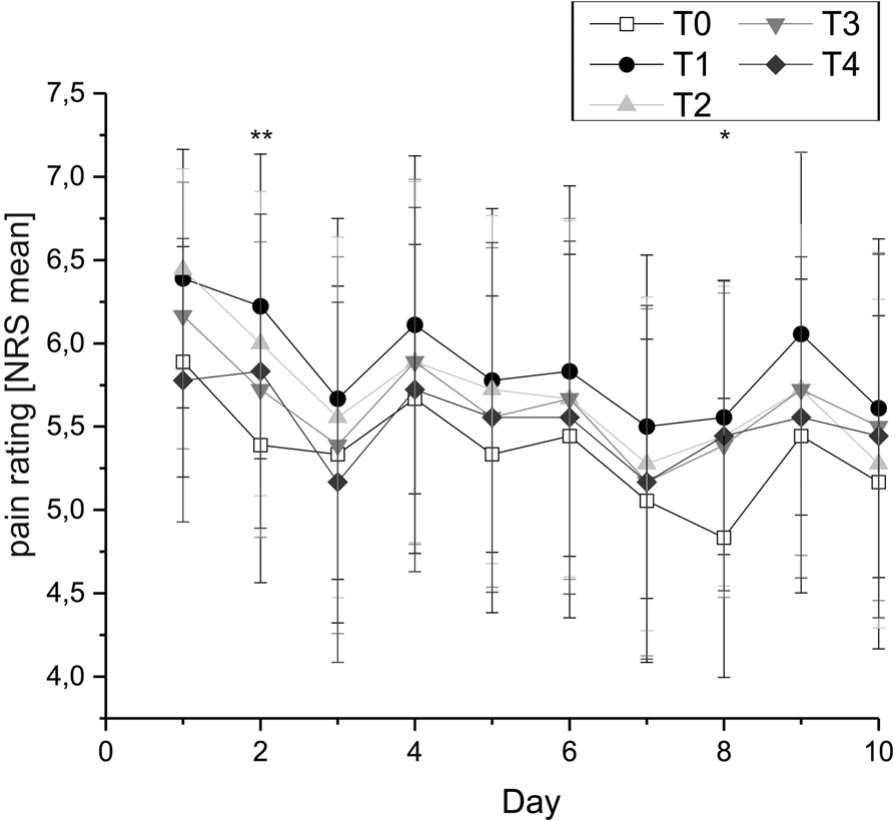
Pain ratings (group mean; raw NRS data) on every day of the investigation (10 days) for all points of assessing pain intensity (T0-T4)

**Fig. 6:**
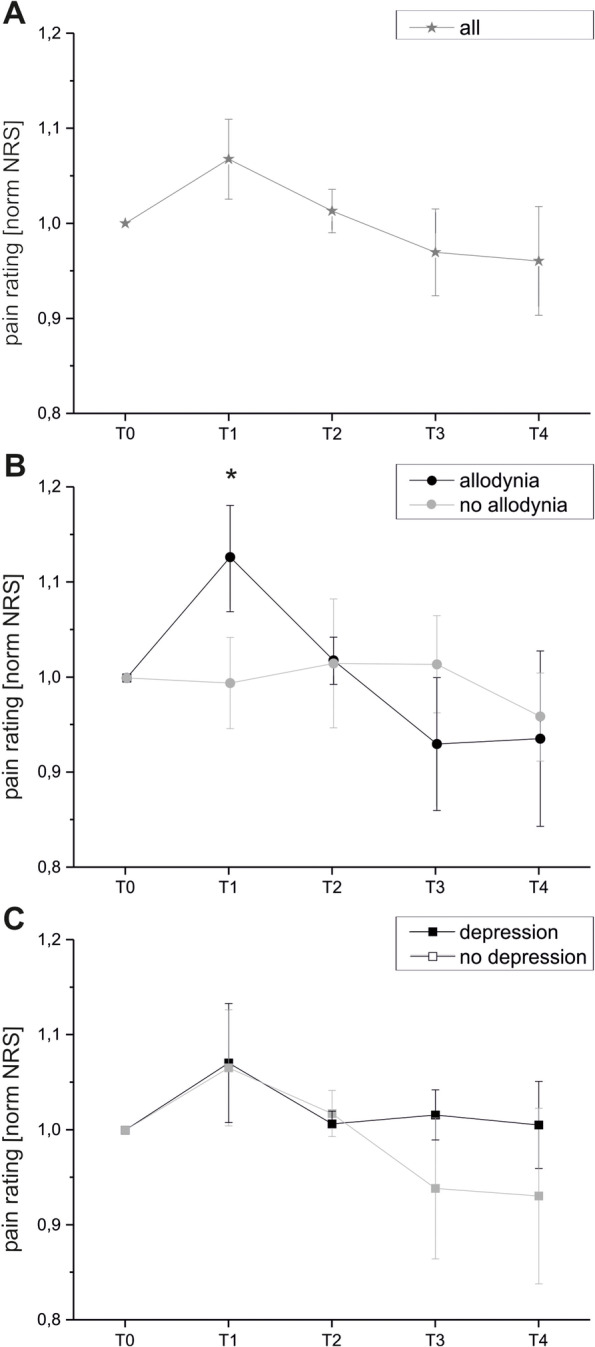
Pain rating at T0-T4 (normalized NRS data) for **A**: all CRPS patients (star) and the following subgroups: **B**: CRPS patients with and without allodynia (circles) and **C**: CRPS patients with and without depressive symptoms (squares)

Pain ratings were highest during CT stimulation (T1). Only on day 2 and day 8 of the investigation, this effect reached level of significance (NRS ratings: day 2: T0 mean 5.39, SD 2.47, SEM 0.82; T1 mean 6.22, SD 2.74, SEM 0.91; p 0.01. Day 8: T0 mean 4.83, SD 2.51, SEM 0.84; T1 mean 5.72, SD 2.93, SEM 0.98; p 0.012). For further details see Figs. 5 and 6A.

Patients with allodynia showed significantly higher pain intensities at T1 (moment of CT stimulation; p 0.029). There was no change of pain intensity over time. For further details, see Fig. 6B.

There were no differences of pain intensity comparing patients with and without depressive symptoms (e.g. NRS at T1 patients with “depression”: mean 5.71, SD 3.62, SEM 1.48, without “depression”: mean 4.17, SD 2.55, SEM 1.47; n.s.). For further details, see Fig. 6C.

Regarding the trend of pain intensity over time (day 1 till day 10), we observed significant lower pain intensities at T2 (30 min after CT stimulation) at day 10 compared to ratings at day 1 (day 1 mean 6.44, SD 1.81, SEM 0.60; day 10 mean 5.28, SD 2.96, SEM 0.99; p 0.048). This pain reduction could not be observed 1 and 2 h after CT stimulation (T3, T4) anymore. All the other points of pain ratings (at T0, T1, T3 and T4) showed no significant differences on the first and last day of the investigation. See Fig. 5.

Overall comparison of pain ratings at T0 to T4 showed a negative trend to lower pain ratings while the investigation (n.s.). See Fig. 6.

## Discussion

CT targeted touch is insufficient to reduce pain intensity in CRPS. This finding is independent from the presence of allodynia. In contrast, repetitive CT fiber stimulation increased thermal pain thresholds. Therefore, pain modulating capacities of CT fibers might exist even in chronic pain states but they are too weak to reduce intensity of chronic pain.

### Pain modulation

CT stimulation by slow brush stroking did not reduce overall pain intensity in CRPS patients. Previous studies showed that intact CT fibers can modulate experimental pain [19, 31]. However, in chronic pain, like CRPS, the pain modulating capacities of CT fibers might be simply too weak to reduce pain. In SFN patients with neuropathic pain, CT targeted touch was unable to alter the perceived intensity of experimental heat pain [19]. In these patients, thermal detection thresholds as well as skin biopsies showed pathological values indicating that CT fibers are impaired. In our CRPS patients, thermal detection thresholds were within normal range at the beginning, indicating that the small nerve fibers, e.g. CT fibers, were intact. At the very beginning of our trial, thermal pain thresholds in CRPS were lower indicating heat and cold hyperalgesia on the affected limb as a sign of hyperexcitability of nociceptors [6, 32, 33]. Hyperalgesia to heat is believed to be a distinct sign of peripheral sensitisation [34] caused by e.g. elevation of inflammatory mediators. Huge and coworkers also observed heat and cold hyperalgesia in acute CRPS patients showing inflammatory signs (reddening, oedema, sweating, heat) to a much greater extent than in chronic CRPS patients [33] underpinning this assumption. At the end of the trial thermal pain thresholds increased, but the actual pain intensity did not decrease.

Furthermore, thermal detection thresholds were reduced at the end of our trial, possibly pointing to a pathology of small diameter nerve fibers. Then again, the combination of hypalgesia to thermal pain and reduced thermal detection might be attributed to a refractoriness of e.g. TRP channels [35, 36]. Since these are above all thermoreceptive and chemoreceptive, they might not mediate nociceptive pain, so that the overall pain intensity remained constant.

All patients underwent also multimodal pain therapy, therefore it could be postulated that other therapies influenced our results. However, it appears obsolete that multimodal pain therapy would only act on experimental induced heat and cold pain. Auxiliary, Maher and coworkers investigated the effect of ketamine infusions and opioids on QST profiles and did not find significant effects on thermal pain thresholds [37]. Other investigations, that especially focused on changes of QST parameters in CRPS patients showed mild changes of pressure pain threshold (PPT) and wind up ratio (WUR) after some month of multimodal therapy. These parameters have been excluded preliminary from our analysis. Moreover, we already showed that CT targeted touch reduces acute experimental heat pain.

Therefore we postulate that repetitive CT targeted touch reduced experimental induced pain in accordance to previous studies on healthy individuals (Habig et al., 2017 [19]).

### Pleasantness rating

As expected, stroking on hairy skin was felt significantly more pleasant than on glabrous skin in healthy subjects. This can be reasoned by the histological distribution of CT fibers, which are known conductors of pleasant touch [15] and only exist in hairy skin but not in glabrous skin [8, 9].

In CRPS patients, CT stimulation on the affected limb was less pleasant and there was no difference between stroking hairy or glabrous skin. Hence, CT fibers might lose their ability to mediate pleasant aspects of touch in chronic pain states. Some of our patients suffered from allodynia and, as expected, these patients felt stroking as less pleasant on the affected limb. However, our results cannot be simply explained by the presence of neuropathic pain since our CRPS patients without allodynia also rated CT touch as less pleasant on hairy skin of the affected limb than healthy controls. Furthermore, the observation that patients with allodynia rated pleasantness of CT touch as not different on glabrous or hairy skin of the affected limb, might indicate a more relevant loss of CT fiber function in patients with allodynia. On the other hand Nagi and coworkers demonstrated, that CT afferents in hairy skin mediated experimental vibration induced allodynia in healthy individuals [38]. Liljencrantz supported this finding with the heat/capsaicin model and a special group of patients with a complete loss of A beta afferents [12]. These observations underpin our thesis, that CT afferents change their pain modulating capacities in chronic pain states.

Where does the pain modulating CT influence take place? Opioid blockade has been found to increase pleasantness of CT targeted touch in healthy individuals but not in chronic pain patients suffering from fibromyalgia [39], indicating a modulation of CT fiber function on a central level. Additionally, a recent work shows that CT targeted touch in patients with fibromyalgia show the same bold responses as healthy controls in the right posterior insula, but have a different activation pattern (activation during pain rating and deactivation during pleasantness rating in patients with fibromyalgia; vice versa in healthy controls), suggesting an intact stimulus perception but a central modification of stimulus evaluation in chronic pain patients [40]. It might be speculated that social aspects of gentle touch become less important in chronic pain states. This phenomenon could be explained in the light of neglect-like findings in CRPS [41, 42]. Moreover, neuroplastic central changes that have been discovered in CRPS [43–46] might alter central processing of CT fiber information. Another possible explanation is that the feeling of chronic pain simply overrules ‘normal’ CT fiber function [47, 48]. Also emotional confounders like depressive symptoms can influence the evaluation of pleasant touch negatively, as shown above (Fig. 4). Patients with concomitant depressive symptoms rated CT touch as less pleasant even on glabrous skin, where CT afferents are absent. Further investigations need to be added to explain pain modulation (decrease of pain intensity and allodynia) by CT afferents in chronic pain patients.

### Limitations

CRPS patients are a heterogenous patient group especially regarding QST parameters [6]. We investigated a small number of patients with different age, medical history and duration of the disease; therefore, subgroup analysis can be error prone.

Since all patients underwent multimodal pain therapy during the experiment, including drugs targeting the central nervous system, we cannot exclude effects of these therapies on our results. Effects of multimodal pain therapy on QST parameters have only been investigated for the long term (6 month, 8 years).The relevant surveys found only mild changes (PPT and WUR reduced) [49, 50], which have been excluded from our analysis. Reversion of cortical reorganization has been shown after 1–6 month [51] or one year [52] of therapy, thus it is not likely, that it has already occurred after two weeks.

Drugs against neuropathic pain (calcium channel blockade; antidepressants; local anaesthetics) act systemic, but we only found changes of QST between both points of assessment at the affected leg, which makes a short term influence on QST parameters of these substances implausible.

We did not perform skin biopsies to determine the intraepidermal nerve fiber density in both limbs, therefore we have no histological verification of the presence of small diameter nerve fibers, including CT afferents.

Furthermore, it would have been interesting to investigate different stroking velocities to see, if e.g. Abeta fiber stimulation leads to different results. However, we only used CT fiber optimal stroking velocity to focus on the main goal of the study (to evaluate the impact of CT stimulation on chronic pain in CRPS) and to avoid interference of other sensory stimuli with CT touch.

## Conclusion

CT-stimulation did not reduce pain intensity in CRPS patients and was less pleasant on the affected limb. Therefore, we present evidence that CT afferents lose their pain modulating properties in a chronic pain condition. It appears possible that CT-afferents might contribute to chronic pain in CRPS. Additionally, CT afferents seem to be involved in allodynia.

## Data Availability

The datasets used and/or analysed during the current study are available from the corresponding author on reasonable request.

## Abbreviations

ADS: German general depression score, “Allgemeine Depressionsskala”
CDT: Cold detection threshold
C-LTMR: C-low threshold mechanoreceptive
CPT: Cold pain threshold
CRPS: Complex regional pain syndrome
CT: C tactile, C touch
DMA: Dynamic mechanical allodynia
HPT: Heat pain threshold
IENFD: Intraepidermal nerve fiber density
IQR: Interquartile range
MPS: Mechanical pain sensitivity
MPSS: Mainz Pain staging system
MPT: Mechanical pain threshold
MRI: Magnetic resonance imaging
NRS: Numeric rating scale
PDI: Pain disability index
PESa: Pain experience scale affective
PESs: Pain experience scale sensory
PHS: Paradoxical heat sensations
PPT: Pressure pain threshold
QST: Quantitative sensory testing
SD: Standard deviation
SEM: Standard error of mean
SFN: Small fiber neuropathy
TRP: Transient receptor potential
TSL: Thermal sensory limen
WDT: Warm detection threshold
